# Changes in lipids and inflammation in adults with super-refractory status epilepticus on a ketogenic diet

**DOI:** 10.3389/fmolb.2023.1173039

**Published:** 2023-10-23

**Authors:** Alex M. Dickens, Tory P. Johnson, Santosh Lamichhane, Anupama Kumar, Carlos A. Pardo, Erie G. Gutierrez, Norman Haughey, Mackenzie C. Cervenka

**Affiliations:** ^1^ Turku Bioscience Centre, University of Turku and Åbo Akademi University, Turku, Finland; ^2^ Department of Chemistry, University of Turku, Turku, Finland; ^3^ Department of Neurology, Johns Hopkins University School of Medicine, Baltimore, MD, United States; ^4^ Department of Psychiatry, Johns Hopkins University School of Medicine, Baltimore, MD, United States

**Keywords:** lipid, inflammation, status epilepticus, epilepsy, ketogenic diet

## Abstract

**Introduction:** This study aims to test the hypothesis that increased ketone body production resulting from a ketogenic diet (KD) will correlate with reductions in pro-inflammatory cytokines and lipid subspecies and improved clinical outcomes in adults treated with an adjunctive ketogenic diet for super-refractory status epilepticus (SRSE).

**Methods:** Adults (18 years or older) were treated with a 4:1 (fat: carbohydrate and protein) ratio of enteral KD as adjunctive therapy to pharmacologic seizure suppression in SRSE. Blood and urine samples and clinical measurements were collected at baseline (*n* = 10), after 1 week (*n* = 8), and after 2 weeks of KD (*n* = 5). In addition, urine acetoacetate, serum *β*-hydroxybutyrate, lipidomics, pro-inflammatory cytokines (IL-1β and IL-6), chemokines (CCL3, CCL4, and CXCL13), and clinical measurements were obtained at these three time points. Univariate and multivariate data analyses were performed to determine the correlation between ketone body production and circulating lipids, inflammatory biomarkers, and clinical outcomes.

**Results:** Changes in lipids included an increase in ceramides, mono-hexosylceramide, sphingomyelin, phosphocholine, and phosphoserines, and there was a significant reduction in pro-inflammatory mediators, IL-6 and CXCL13, seen at 1 and 2 weeks of KD. Higher blood *β*-hydroxybutyrate levels at baseline correlated with better clinical outcomes; however, ketone body production did not correlate with other variables during treatment. Higher chemokine CCL3 levels following treatment correlated with a longer stay in the intensive care unit and a higher modified Rankin Scale score (worse neurologic disability) at discharge and 6-month follow up.

**Discussion:** Adults receiving an adjunctive enteral ketogenic diet for super-refractory status epilepticus exhibit alterations in select pro-inflammatory cytokines and lipid species that may predict their response to treatment.

## 1 Introduction

Status epilepticus (SE) is the second most frequent neurologic emergency worldwide (DeLorenzo et al., 1996; Knake et al., 2001; Rossetti and Lowenstein, 2011). Super-refractory status epilepticus (SRSE) (Hocker et al., 2014), which persists despite 24 h of aggressive intervention, carries an in-hospital mortality rate above 40% (Jayalakshmi et al., 2014; Strzelczyk et al., 2017). The ketogenic diet (KD) is a high-fat, carbohydrate-restricted diet that shows promise for treating SRSE, whose mechanism involves fatty acid metabolism (Cervenka and Kossoff, 2013; Wusthoff et al., 2009; Cervenka et al., 2011; Nam et al., 2011; Thakur et al., 2014; Peng et al., 2019; Strzelczyk et al., 2013; Lin et al., 2015). A study using a 4:1 ratio of KD in 15 adults with SRSE found that 73% had resolution of SRSE, and more recent studies have replicated these findings (Francis et al., 2019; Park et al., 2019; Peng et al., 2019).

The exact mechanisms by which KD halts status epilepticus remain under investigation. During prolonged status epilepticus, systemic inflammation leads to loss of blood–brain barrier integrity, leakage of neurotoxic serum proteins into the brain, and loss of inhibitory interneurons, all promoting further neuronal hyperexcitability (Vezzani et al., 2005; Ravizza et al., 2011; Librizzi et al., 2012; Devinsky et al., 2013; Vezzani et al., 2013). Specific pro-inflammatory cytokines and chemokines, including IL-1β, IL-6, CCL2, and CCL3, significantly increase with disease progression (De Simoni et al., 2000; Lv et al., 2014; Eriksson et al., 2000; Ravizza and Vezzani, 2006; Foresti et al., 2009; Fabene et al., 2010; Aronica and Crino, 2011; Pernot et al., 2011; Kan et al., 2012; Zhu et al., 2012; Hung et al., 2013). KD reduces inflammation, thereby potentially halting this cyclical progression of neuronal injury and death (Fraser et al., 2000a; Fraser et al., 2000b; Yang and Cheng, 2010; Buy et al., 2014; Dupuis et al., 2015; Schreck et al., 2017).

Another potential mechanism of action of KD may be lipid modulation which occurs when fatty acids are utilized during carbohydrate restriction. Certain lipid subspecies have been shown to be anti-inflammatory, which may also explain the impact of KD on the inflammatory cascade (Dupuis et al., 2015). This study examines the relationship between systemic inflammation, lipid modulation, and clinical outcomes in adults on KD for the management of SRSE. We hypothesized that increased ketone body production would correlate with a reduction in pro-inflammatory cytokines and pro-inflammatory lipid subspecies and improved clinical outcomes.

## 2 Materials and methods

### 2.1 Population and demographics

The patients included in this study have previously been described (Cervenka et al., 2017). In brief, 15 patients with SRSE were recruited into a phase I/II clinical trial, of which 10 were included in this study. The patients were treated with a 4:1 (fat: carbohydrate and protein in grams) ratio of KD upon diagnosis or hospital transfer. Clinical characteristics included participant age, gender, race, history of epilepsy prior to admission, and etiology of SRSE. “Ketosis” was defined as urine acetoacetate ≥40 mg/dL and/or serum *β*-hydroxybutyrate ≥2 mmol/L in order to calculate the time to ketosis as a clinical outcome variable. Aliquots of plasma were collected from the patients repeatedly over time at 1-week intervals when possible. The plasma was stored at −80°C prior to analysis. Due to the low number of samples longitudinally, we categorized the patients into four groups defined as follows:

• Baseline: Blood samples taken prior to the start of the ketogenic diet (*n* = 10)
• Week 1: Blood samples taken after 1 week of following a ketogenic diet (*n* = 8)
• Week 2: Blood samples taken after 2 weeks of following a ketogenic diet (*n* = 5)
• Patients in this study were followed up for 6 months after hospital discharge, and modified Rankin Scale scores were collected for all participants (*n* = 10).

### 2.2 Plasma lipid extraction

The lipids were extracted from the plasma using a modified Bligh–Dyer approach. Throughout the procedure, all steps were performed with glass to avoid extracting lipid-like structures from laboratory plasticware. All solvents used were of ultra-pure HPLC grade. A measure of 30 μL of plasma was gently mixed with 970 mL of ddH_2_O and 2.9 mL of MeOH:DCM mix (2:0.9 v/v) to form a monophasic mixture. The organic fraction contained a mix of the following lipids as internal standards: N-lauroyl-D-erythro-sphingosine (Cer d18:1/12:0, 6 ng/mL), 1,3 (d5)-dihexadecanoyl-glycerol (d5-DAG d16:0/16:0, 12.5 ng/mL), D-galactosyl-β-1,1′-N-lauroyl-D-erythro-sphingosine (GlcCer d18:1/12:0, 3.3 ng/mL), D-lactosyl-β-1,1′-N-lauroyl-D-erythro-sphingosine (LacCer 18:1/12:0, 10.6 ng/mL), 1,3 (d5)-dihexadecanoyl-2-octadecanoyl-glycerol (D-5 TAG 16:0/18:0/16:0, 0.5 ng/mL), cholesteryl-d7 palmitate (cholesteryl-d7 ester 16:0, 30 ng/mL), 1,2-dilauroyl-sn-glycero-3-phosphate (sodium salt) (PA d12:0/12:0, 1,025 ng/mL), 1,2-dilauroyl-sn-glycero-3-phosphocholine (PC 12:0/12:0, 0.2 ng/mL), 1,2-dilauroyl-sn-glycero-3-phosphoethanolamine (PE d12:0/12:0, 1.6 ng/mL), 1,2-dilauroyl-sn-glycero-3-phospho-[1′-rac-glycerol] (PG d12:0/12:0, 200 ng/mL), 1,2-dilauroyl-sn-glycero-3-phospho-L-serine (PS d12:0/12:0), and N-lauroyl-D-erythro-sphingosylphosphorylcholine (SM d18:1/12:0, 0.3 ng/mL); all internal standards were purchased from Avanti Polar Lipids, Inc. (Alabaster, AL). A measure of 1 mL of ddH_2_O and 0.9 mL of DCM were added to the mixture, and the sample was briefly vortexed, resulting in a biphasic mixture. This mixture was allowed to stand for 30 min on ice prior to centrifugation (10 min, 3,000 × g, 4°C). The organic phase was then removed and stored at −20°C until the mass spectrometry analysis.

### 2.3 Mass spectrometry analysis

Prior to the mass spectrometry analysis, 1 mL of the organic extract was evaporated to dryness under a stream of nitrogen and re-suspended in the running solvent (250 μL, MeOH:DCM, 1:1, containing 5 mM NH_4_CH_3_). The running solvent also contained 5 mg/mL of ceramide C17:0 as a further internal standard to monitor instrument performance over time independently of the extraction process. The concentrations of the lipids were then measured using an MS/MS^ALL^ experiment in positive mode on a TripleTOF™ 5600 (SCIEX, United States) mass spectrometer. This is a data-independent acquisition mode, where the first quad steps through all masses from 200 to 1,200 Da in 1 Da steps. The selected precursor ion was then fragmented and quantified by TOF with a scan range of 100–1,500 Da. The accumulation time was set to 450 ms, and the data were acquired using Analyst TF 1.7 software (SCIEX, United States). The mass calibration was performed after the run based on the internal standards within the sample. The sample (50 µL) was continually infused into the spectrometer at a constant flow rate (5 μL/min) using a LC-20AD pump and SIL-20AC XR HPLC system (Shimadzu, United States). The source parameters of the mass spectrometer were set up as follows: ion source gases 15 psi (GSI) and 20 psi (GS2), curtain gas 30 psi, temperature 150°C, positive ion spray voltage +5,500 V, declustering potential at 80 V, and collision energy at 10 V. All samples were run in duplicate.

### 2.4 Identification of lipids

In order to build a targeted method to extract out the identified lipids from each sample, a pooled sample was run eight times using the same mass spectrometry method. The lipids were then identified based on their fragmentation patterns using LipidView software (SCIEX, United States). Each lipid species identified had to appear in seven out of the eight replicates and have a coefficient of variation below 20% in order for it to be included in the targeted lipid list. The resultant lipid list was then used to create a targeted method for extracting these specific lipids in LipidView software (SCIEX, United States). The method was then applied to all the samples using MultiQuant v3.0 software (SCIEX, United States). The lipid intensities were corrected to their relevant class based on internal standards. If the duplicate samples varied by more than 30% in intensity, then the samples were re-run. For statistical analysis, zero intensities were imputed by dividing the lowest-intensity lipid by a factor of 1,000.

### 2.5 Cytokine and chemokine quantification

The concentrations of IL-1β, IL-6, TNFα, CCL3, CCL4, and CXCL13 in serum were measured serially in six participants by microbead multiplexed assays (Luminex^®^) according to the manufacturer’s instructions. Only those participants who were not treated with concomitant immunosuppression were included in cytokine and chemokine analyses. Cytokine levels were measured prior to initiation of the ketogenic diet and at 1 and/or 2 weeks after diet initiation, when possible.

### 2.6 Statistical analysis

The univariate analysis of lipid changes over time was assessed using a Wilcoxon rank test with multiple test corrections. PLS-DA multivariate statistical analysis was performed using the PLS Toolbox v 8.5 (Eigenvector Labs, United States) for MATLAB 2018a (MathWorks, United States). The PLS-DA models were cross-validated with a leave-one-out approach due to the low number of samples. Block variance scaling was used to scale the multiblock data. Multiblock-ANOVA–simultaneous component analysis (ASCA), a multivariate extension of ANOVA, was performed to allow interpretation of the variation-induced categorical factors, including case (baseline *versus* week 1), sex, race, and history of epilepsy prior to admission. This multivariate analysis was also performed using PLS Toolbox 8.2.1 (Eigenvector Research Inc., Manson, WA, United States) in MATLAB 2017b (MathWorks, Inc., Natick, MA, United States).

All univariate analysis and resultant bar graphs were performed in Prism v 7.04 (GraphPad, United States). For the four group comparisons, one-way ANOVA tests were used, and multiple comparisons were corrected using the Benjamini and Hochberg false discovery rate (FDR). Significant changes were defined as q-values less than 0.05. Spearman’s correlation coefficients were calculated using the statistical toolbox in MATLAB 2017b, and *p*-values <0.05 (two-tailed) were considered significant for the correlations. The individual Spearman’s correlation coefficients (R) were illustrated in a circular correlation plot using the “corrplot” package for the R statistical programming language. For any missing values, the half minima of that variable were used to impute the value.

Cytokine and chemokine results are presented as index to control with the pre-ketogenic baseline measure set to 100%. According to previously described analyses strategies, if a measurement was below the limit of detection for a patient, it was assigned the value of half the limit of detection and if more than one measurement was below the limit of detection for the same patient, that patient was excluded from analysis (Beal, 2001). Measurements were assessed for statistical outliers, and outliers (patient 8 CCL3 measurement, patient 1 CCL4 measurement, patient 10 TNF-α measurement, and patient 3 CXCL13 measurement) were not included in analyses but are shown in the figures. Decreases in cytokine or chemokine concentrations from baseline after initiation of the ketogenic diet were compared using a one-tailed paired *t*-test with *p* < 0.05 considered statistically significant. Statistical analyses were performed using GraphPad software version 8.3.1.

### 2.7 Model-based clustering

Clustering of the lipidomic data was implemented using the MCLUST R package (version 5.4.5). MCLUST is a model-based clustering method, where the model performances are evaluated by the Bayesian information criterion. Generally, the model with the highest Bayesian information criterion is selected.

## 3 Results

### 3.1 Circulating lipids and cytokines

**FIGURE 1:**
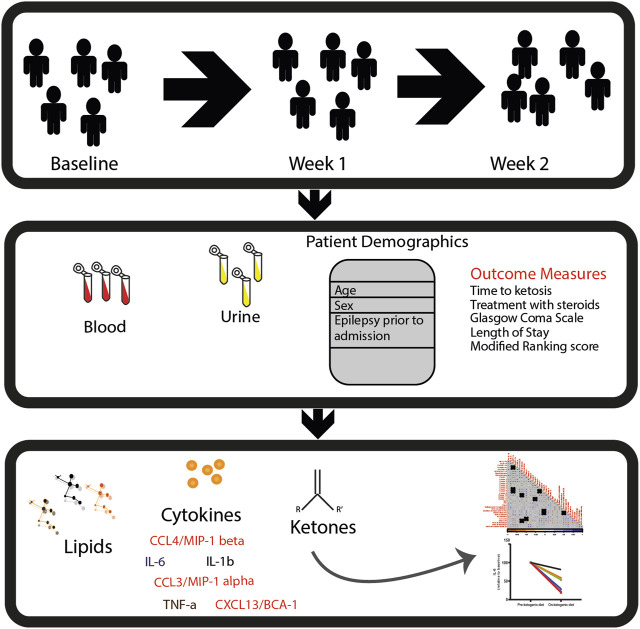
Study design. In this study, patients with SRSE were recruited into a phase I/II clinical trial and treated with a 4:1 (fat: carbohydrate and protein in grams) ratio of ketogenic diet. Samples (blood and urine) and clinical measurements were collected at baseline: prior to KD (*n* = 10), week 1: after 1 week of KD (*n* = 8), and week 2: after 2 weeks of KD (*n* = 5). Ketosis was confirmed by urine acetoacetate ≥40 mg/dL and/or serum *β*-hydroxybutyrate ≥2 mmol/L. Lipidomics, cytokine, and chemokine measurements were performed in longitudinal settings. Then, univariate and multivariate data analyses were performed to determine the potential impact of diet on circulating lipids and inflammatory biomarkers.

There were a total of 487 lipids detected and identified in the serum of study participants. In addition, we also measured a panel of six cytokines and chemokines from the same samples. See Figure 1 for the overall experimental design.

### 3.2 Lipid changes with the ketogenic diet

Using a univariate approach, we identified a total of nine lipids that changed in abundance from baseline to week 1 and 29 lipids that changed in abundance by week 2, with q = 0.1 (see Table 1). Unknown triacylglycerol (TAG) species (54:11) and TAG (54:5) consistently increased from baseline to week 1 and from week 1 to week 2.

**TABLE 1:** Lipids which changed at week 1 or week 2.

Lipid	Week 1 Q	Week 2 Q
Unknown TAG species (54:11)	0.090	0.094
TAG (58:7)	0.090	0.236
TAG (54:4)	0.090	0.134
TAG (54:5)	0.090	0.070
TAG (54:4)	0.090	0.134
TAG (54:3)	0.090	0.276
PS_O (34:6)	0.090	0.386
HexCer (30:0; 2)	0.090	0.457
TAG (58:5)	0.090	0.276
TAG (54:4)	0.110	0.078
TAG (54.5)	0.110	0.081
TAG (56.4)	0.110	0.081
TAG (56.5)	0.110	0.092
TAG (56:7)	0.116	0.022
TAG (56:5)	0.116	0.078
HexCer (26:1; 3)	0.116	0.081
Unknown TAG species (54.9)	0.116	0.092
Unknown TAG species (54:10)	0.126	0.078
TAG (56:6)	0.186	0.022
TAG (56.4)	0.186	0.081
TAG (56:7)	0.199	0.015
TAG (56.6)	0.207	0.081
Unknown TAG species (54:11)	0.222	0.081
Unknown TAG species (52:11)	0.229	0.015
TAG (56:5)	0.250	0.078
HexCer (40:0; 3)	0.265	0.022
Unknown TAG species (50:10)	0.277	0.034
Cer (42:1; 3)	0.280	0.097
PS (38:0)	0.305	0.081
TAG (56:5)	0.308	0.078
Unknown TAG species (52:10)	0.310	0.078
Unknown TAG species (54:4)	0.322	0.078
TAG (48:3)	0.336	0.081
TAG (52:6)	0.353	0.081
Unknown TAG species (52:10)	0.393	0.078
Unknown TAG species (52:10)	0.396	0.081

**FIGURE 2:**
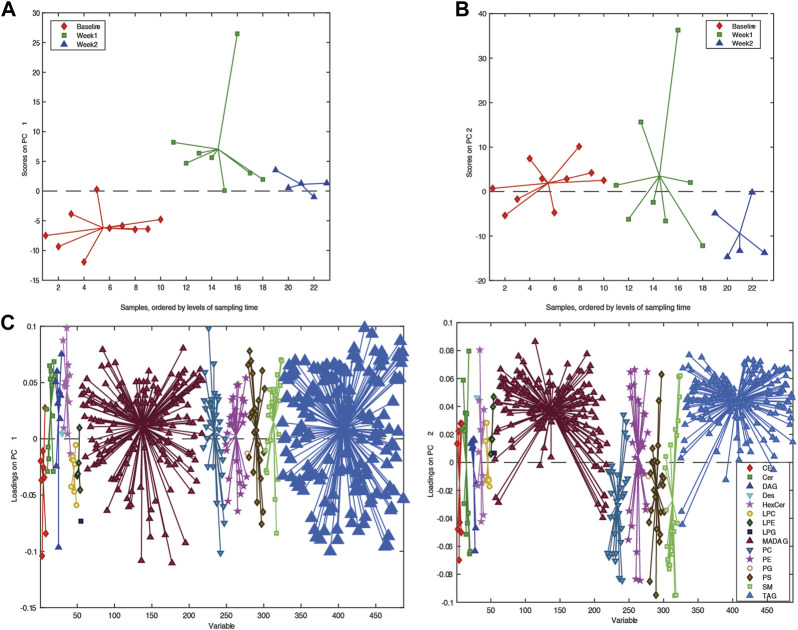
Overview of the lipidome in the longitudinal samples. **(A)** PCA score plots. **(B)** PC1 score plot based on ASCA. These scores represent the lipidomic dataset arranged according to time of sampling in the PCA score plot. Here, each sample is represented by a point and colored according to the time of sample collection. Samples with similar scores are clustered together. **(C)** Corresponding PC1 loading plot. The loadings explain the pattern shown in the score plot, which provides the means to interpret the class-specific lipid alterations related to KD.

Building an ASCA model using the individual lipidomic data from available samples, we observed a clear drift in the multivariate models (Figure 2A), suggesting that there was a clear change in the serum lipid content after participants were placed on a ketogenic diet that persisted over time. To further explore this change in serum lipid content over time, we utilized an ANOVA–simultaneous component analysis (ASCA) multivariate model. This model can determine the effects of different independent variables, such as time within the data, in a manner similar to a standard two-way ANOVA. The ASCA modeling (Figure 2B) showed a distinct effect of time on ketogenic diet (effect = 12.5%) and gender (6.3%). Examination of the loadings of the first principal component for the factor of time on the ketogenic diet demonstrated that several classes of lipids increased over time, including ceramides, mono-hexosyl ceramides, sphingomyelins, phosphocholines, and phosphoserines (Figure 2C).

**FIGURE 3:**
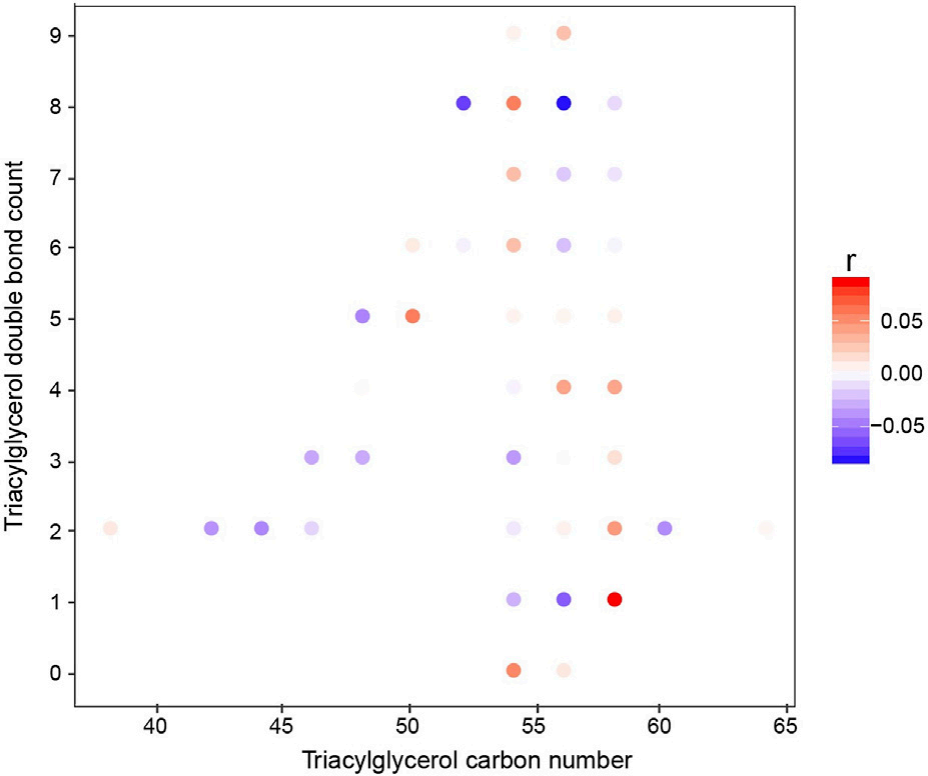
Correlation of individual TGs with the study week (baseline to week 2). The *x*-axis represents the acyl carbon number, and the *y*-axis represents the acyl double-bond count. The dots are colored according to the Spearman’s coefficient.

In the univariate model, there was a clear change in the triglyceride species. Therefore, we plotted how these changed over time. Interestingly, the short-chain unsaturated lipids decreased in concentration while on the diet, whereas the longer and more saturated compounds increased (Figure 3). These shorter-chain saturated TAGs result from *de novo* lipogenesis, suggesting that the ketogenic diet is actively suppressing this metabolic pathway. The larger unsaturated species result from the diet, and this is to be expected given the diet these patients are on.

### 3.3 Serum cytokine changes over time

**FIGURE 4:**
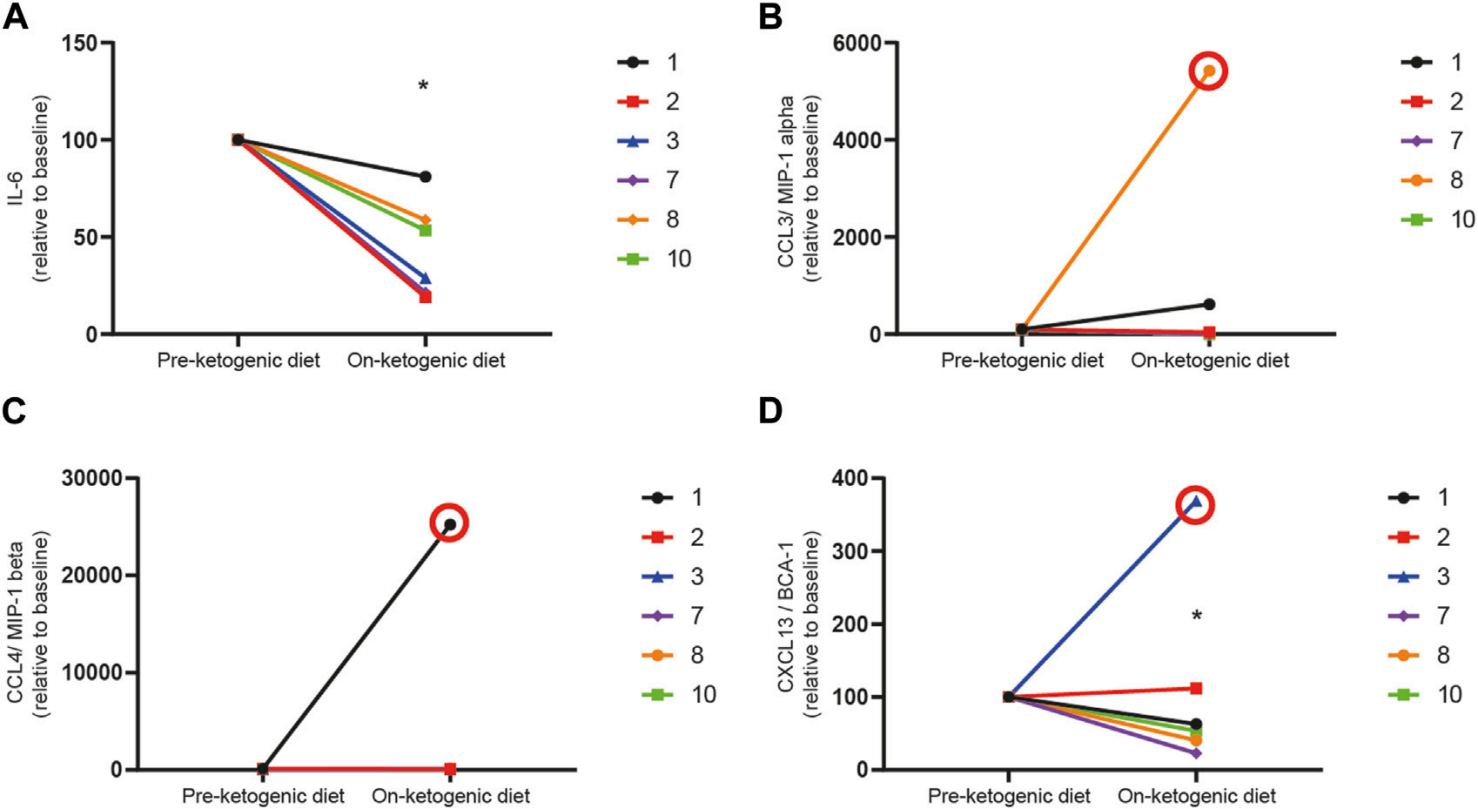
The ketogenic diet reduces pro-inflammatory cytokines in patients with refractory status epilepticus. Serum levels of **(A)** IL-6, **(B)** CCL3, **(C)** CCL4, and **(D)** CXCL13 were measured before patients initiated the ketogenic diet (pre-ketogenic diet) and 1–2 weeks after diet initiation (on-ketogenic diet). Data are expressed as the index of each cytokine with each patient baseline set to 100 (arbitrary units). Data were analyzed by a one-way paired *t*-test. There was a significant reduction in IL-6 (*p = 0.0047) and CXCL13 (*p = 0.025) in patients on the diet as compared to their baseline samples but not in CCL3 (*p* = 0.0854) or CCL4 (0.1124). Samples found to be statistical outliers are circled in red and are not included in analyses.

Two cytokines, IL-1β or TNFα, were excluded from further analysis as only two participants had measurable levels above the limits of detection for the assay. The concentrations of IL-6 (*n* = 5), CCL3 (*n* = 5), CCL4 (*n* = 6), and CXCL13 (*n* = 6) were reliably measured in paired samples of participants, and concentrations of each cytokine were normalized to individual participant at baseline and compared after 1–2 weeks on the ketogenic diet (Figure 4). Despite the small number of samples with detectable levels of cytokines pre- and post-treatment, there was a significant reduction in IL-6 (Figure 4A) at 1–2 weeks post-ketogenic diet compared to baseline (mean ± SD, pre-diet 100 ± 0 *versus* post-diet 43.8 ± 24.7, *p* = 0.0013, one-way paired *t*-test). No differences in CCL3 (Figure 4B) (mean ± SD, pre-diet 100 ± 0 *versus* post-diet 166.1 ± 302.6, *p* = 0.3458, one-way paired *t*-test) or CCL4 (Figure 4C) (mean ± SD, pre-diet 100 ± 0 *versus* post-diet 85.87 ± 22.02, *p* = 0.1124, one-way paired *t*-test) were detected although previously found to decrease in one preclinical study of the ketogenic diet (Kim do et al., 2012). An outlier (patient 3) was detected in measurements for CXCL13 (Figure 4D), and therefore, this patient was excluded from analyses. There was a significant reduction in CXCL13 after the ketogenic diet as compared to pre-diet (mean ± SD, pre-diet 100 ± 0 *versus* post-diet 58.36 ± 33.56, *p* = 0.025, one-way paired *t*-test).

### 3.4 Associations between serum lipids and cytokines

**FIGURE 5:**
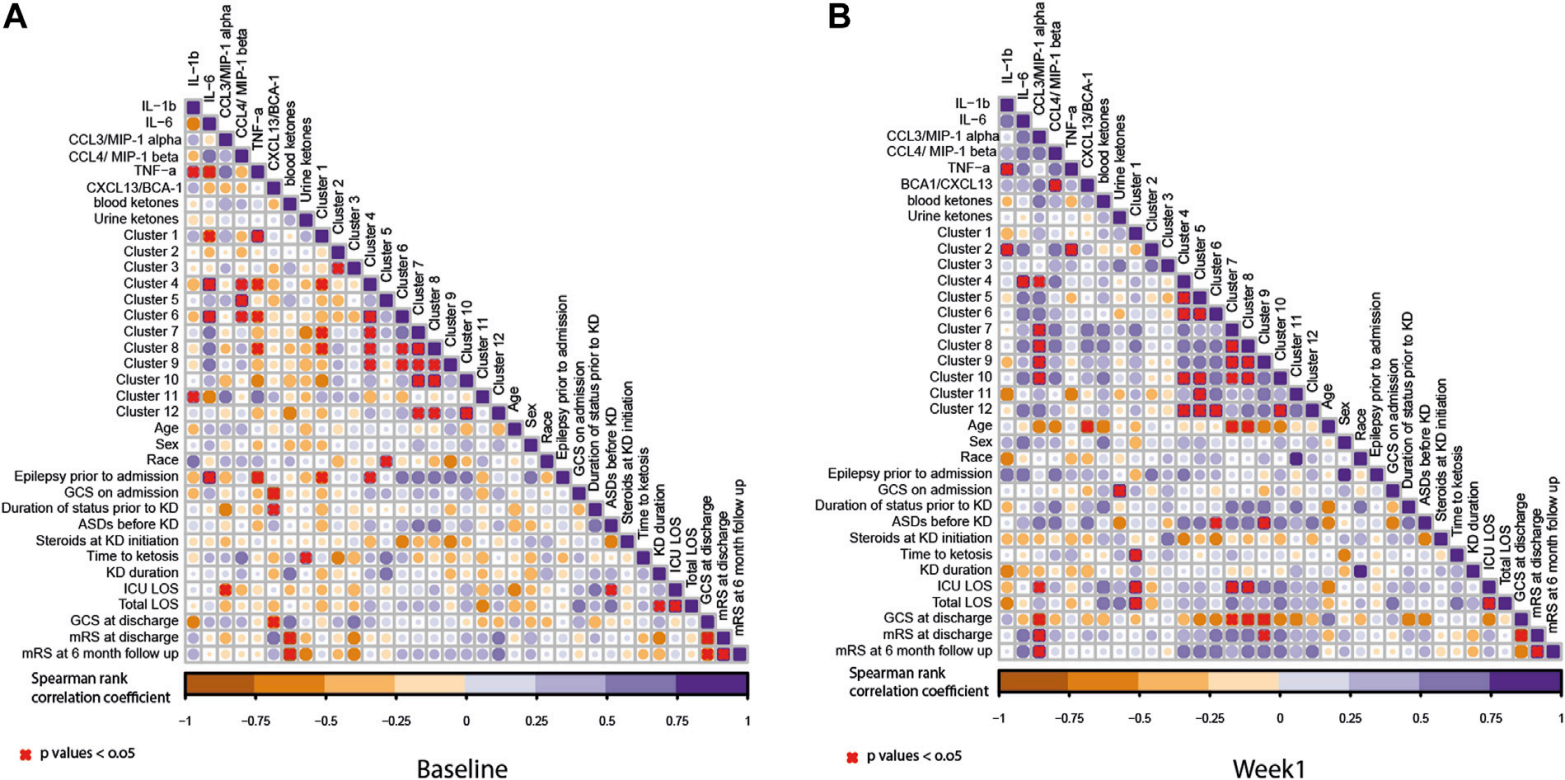
Correlation coefficients illustrated by a heat map. The correlation analysis was performed between lipid clusters and other measurements including cytokine measurements, ketones, patient demographics, and outcome measure as listed in Figure 1. **(A)** Baseline and **(B)** week 1 samples. The circles in the plot represent the Spearman’s correlation coefficient between the measurements. Here, the purple gradient represents the strength of the positive correlation, while the dark orange gradient indicates the strength of the negative correlation (red X denotes the *p*-values < 0.05). The size of the circle represents the strength of the correlation coefficient. The bigger circle represents a higher correlation coefficient.

Due to the large number of lipids detected in the serum and the high degree of cross-correlation associated with lipidomic data, we decided to reduce the dimensionality of the data using Bayesian clustering, which has previously been used for lipid datasets. This technique clusters the lipids, which behave in a similar fashion, together. As expected, the lipids were largely clustered based on their class. When plotting the average lipid concentration for each cluster, only a few clusters showed a change over time. These clusters were cluster 3 (*p* = 0.0445) and cluster 12 (*p* = 0.0089). The lipids that are contained in each cluster are shown in Table 2. Lipid cluster 3 contained a broad species of lipids, but it primarily contained lipids that were related to inflammation, such as Cer, SM, and oxidized lipids. Lipid cluster 12 contained large unsaturated TAGs, which is a result of the diet. When looking at the association between lipids, cytokines, and clinical characteristics, there was a high degree of correlation between the lipid clusters, as expected (Figures 5A, B).

**TABLE 2:** Details of lipids contained in each cluster.

Cluster	Size	Lipid types
Cluster 1	92	Cer, LPC, PC, CE, TAG, DAG PS, PS-O, and SM
Cluster 2	10	LPC and LPE
Cluster 3	69	Cer, LPC, PC, CE, PC, PC-O, PE, PS, and TAG
Cluster 4	61	Cer, DAG, TAG and PE
Cluster 5	55	PE, DAG, Cer, PE-O, and TAG
Cluster 6	39	TAG
Cluster 7	31	TAG
Cluster 8	32	TAG
Cluster 9	34	TAG
Cluster 10	30	TAG
Cluster 11	9	TAG
Cluster 12	25	TAG

At baseline, there were positive correlations between lipid clusters 4 and 6 and IL-6 and CCL3. Lipid cluster 4 contained ceramides and sphingomyelins, as well as some diacylglycerols (DAGs), TAGs, and unknown TAG species. Lipid cluster 6 contains predominantly unknown TAG species and TAGs. Additionally, there was a positive correlation with lipid cluster 5 and CCL4. There were further positive correlations observed between IL-1β and lipid cluster 11 and cluster 1 and TNFα. There was also a negative association between TNFα and lipid clusters 4, 5, and 8. Furthermore, there was a negative association between lipid cluster 1 and IL-6. At baseline, the only lipid cluster that positively correlated with the clinical features was lipid cluster 4. This lipid cluster was associated with a history of epilepsy prior to admission for SRSE. More information about the other lipid classes is shown in Table 2. After 1 week of the ketogenic diet, there was an even stronger correlation between the lipid clusters, suggesting that the diet may alter lipid metabolism in patients with SRSE. Interestingly, the patterns observed with the cytokines changed after diet initiation. Lipid cluster 2 is positively associated with IL-1β and TNFα. Lipid cluster 2 contains lyso-lipids, which have been shown to be altered in a pro-inflammatory state (Frasch and Bratton, 2012). The other significant associations between lipids and cytokines were all positive and linked lipid cluster 4 with IL-6 and CCL3. CCL3 was also associated with lipid clusters 7, 8, 9, and 10 which contain the TAG species.

### 3.5 Lipids and inflammatory biomarkers as predictors of clinical outcomes

**FIGURE 6:**
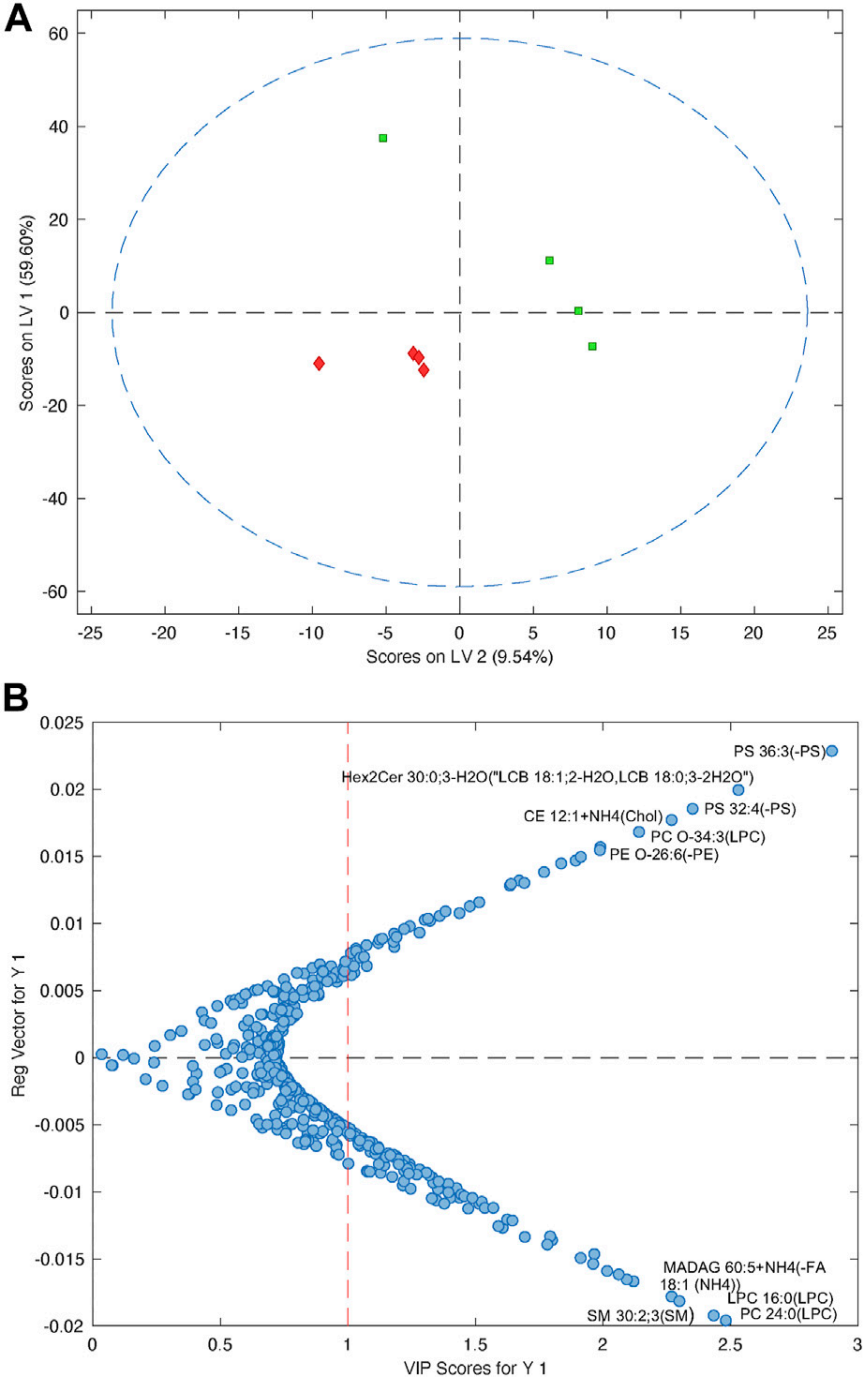
**(A)** PLS-DA model showing the separation of patients with a good outcome (mRS ≤3, green) compared to patients with a worse outcome (mRS ≥4, red). **(B)** Plot showing the variable importance *versus* the regression vector for the lipids that impact the PLS-DA model. Metabolites at the top are increased in patients with poor outcomes, compared to the lipids at the bottom, which are decreased in patients with poor outcomes.

Due to the low number of participants, making predictions on the whole circulating lipidome is very difficult. However, it was possible to build a PLS-DA model using the blood samples taken at week 1, which could differentiate between the participants who recovered with only a mild disability (mRS <3) compared to those who were moderately or more severely disabled or dead (mRS ≥3) (Figure 6A). The cross-validated model had an ROC = 0.56, which is understandable given the very small sample size (*n* = 4 per group). However, from the PLS-DA model, it was possible to identify which lipids cause the separation observed in the model by examining the variable importance scores, which provides a metric on how each lipid contributes to the PLS-DA model.

At baseline (Figure 5), there were very few significant correlations between pro-inflammatory and lipid biomarkers and clinical outcomes. There was a significant negative correlation between the GCS score at discharge and CXCL13 levels prior to diet intervention. In other words, those participants with higher CXCL13 levels prior to diet intervention had poorer neurologic outcomes. There was a strong negative correlation between blood *β*-hyrdroxybutyrate levels at baseline and mRS at discharge and 6-month follow-up. In other words, participants with lower blood ketone levels prior to intervention had a more severe disability or a higher likelihood of death at discharge and at 6 months follow-up.

At 1 week, there were several significant correlations between lipid clusters, CCL3 concentrations, and clinical outcomes. While it was previously noted that CCL3 levels positively correlated with the concentrations of lipid clusters 4, 7, 8, 9, and 10, higher CCL3 levels also correlated with a longer ICU stay and a higher mRS score (worse neurologic disability) at discharge and 6-month follow-up. There was also a significant positive correlation between mRS at discharge and lipid cluster 9. Finally, there was a negative correlation between CCL3 concentrations at week 1; lipid clusters 7, 8, and 9; and GCS scores at discharge.

## 4 Discussion

Super-refractory status epilepticus is a life-threatening disease with no proven treatments. While the ketogenic diet has been used for over one century to manage seizure disorders, it has only been incorporated into the management of refractory and super-refractory status epilepticus in recent years. This study was designed to determine whether treatment with a ketogenic diet and the resultant production of ketone bodies played a role in lipid modulation and reduction in systemic inflammation as potential mechanisms of action for halting seizures in adults with super-refractory status epilepticus.

Prior to the initiation of the ketogenic diet in adults with SRSE, there were several positive correlations between clusters of lipid species and pro-inflammatory cytokine and chemokine levels. There was a specific association between prior history of epilepsy and a lipid cluster that contained ceramides and sphingomyelins, as well as some diacylglycerols, triacylglycerols, and monoalkyldiacylglycerols (unknown TAG species), suggesting that the prior epilepsy could have long-term effects on these lipids or that these changes could be the underlying cause (Mikati et al., 2003). The associations of these lipid clusters with pro-inflammatory cytokines (Wheeler et al., 2009) suggest that they may be markers of the underlying inflammation.

Ceramides are a subclass of sphingolipids that are known to regulate the surface expression of NMDA receptors (Wheeler et al., 2009) and mediate programmed cell death. Ceramides have been shown to increase in the hippocampal tissue of adult rats after kainic acid-induced status epilepticus (Mikati et al., 2003), suggesting a potential role in neuronal injury during status epilepticus. In the study, the levels of ceramides and hexyl ceramides increased over time.

The only biomarker that significantly predicted clinical outcomes prior to KD initiation was the *β*-hydroxybutyrate level. Specifically, higher *β*-hydroxybutyrate levels prior to intervention predicted a lower degree of disability at discharge and 6-month follow-up. In a lipopolysaccharide-induced fever rodent model, researchers observed a lower fever and lower pro-inflammatory cytokines IL-1β and TNFα in animals pre-treated with KD compared to controls, although ketone body measures were not included (Dupuis et al., 2015). The authors concluded that KD may have a neuroprotective effect. In RSE and SRSE, it is well established that early aggressive intervention reduces morbidity and mortality, and therefore, participants who were producing ketone bodies prior to KD (either with fasting or other lipid source pre-diet) may have derived benefits even prior to diet initiation.

There was a significant reduction in the systemic pro-inflammatory cytokine IL-6 and the pro-inflammatory chemokine CXCL13 in adults on KD. If replicated in larger patient cohorts compared to a control group, these findings would be of high clinical significance, as status epilepticus is a pro-inflammatory state and the anti-inflammatory properties of KD may contribute to its effectiveness. Recent studies have shown that the ketone body *β*-hydroxybutyrate directly inhibits NLRP3 inflammasome-mediated production of IL-1β (Youm et al., 2015; Deora et al., 2017; Yamanashi et al., 2017), reducing systemic inflammation in rodents and human monocytes, which may provide an explanation for the reduction in systemic pro-inflammatory cytokines and chemokines observed in this study. However, urine acetoacetate and blood *β*-hydroxybutyrate as well as time to ketosis did not show any direct correlation with clinical outcomes or inflammatory biomarker concentrations in this study. Previous studies examining the relationship between ketone body production and seizure cessation in humans have had mixed results, likely due to the small number of participants (Buchhalter et al., 2017).

In conclusion, higher CXCL13 levels correlated with poorer neurologic outcomes. Recent studies have demonstrated an increase in CXCL13 in rodent models of status epilepticus and postulated that this may play a role in pathogenesis (Li et al., 2017). Lower levels of CCL3 and lipid clusters 7–9 predicted better clinical outcomes at week 1 of intervention with KD.

This study had several important limitations. The number of participants was small, and therefore, few significant correlations could be identified. Blood samples were collected from participants in a single-arm phase I/II study, and a larger randomized controlled phase III clinical trial could better replicate or refute these preliminary findings, which provides a roadmap of potential biomarkers of interest for future investigation. In addition, serial sample collection was not feasible for all participants, and IL-1β and TNFα measures were below the detectable range in most participants. Expected changes in lipids and inflammatory cytokine and chemokine concentrations with the progression of SRSE are unknown, and therefore, which changes may have been a result of KD or ketone body production cannot be determined.

In conclusion, this study provides preliminary evidence that lipid modulation and a reduction in pro-inflammatory biomarkers occur during the administration of a ketogenic diet in adults with super-refractory status epilepticus. Furthermore, lower levels of pro-inflammatory chemokines and certain lipid species predict a better neurologic outcome following KD. Further studies are needed to understand the potential neuroprotective effects of lipid modulation on reduction in inflammation using KD in the setting of refractory status epilepticus and establish the relationship between the modulation of specific lipid species, inflammation, ketone body production, and seizure cessation.

## Data Availability

The original contributions presented in the study are included in the article/Supplementary Material; further inquiries can be directed to the corresponding author.

## References

[B1] Aronica E. Crino P. B. (2011). Inflammation in epilepsy: clinical observations. Epilepsia 52 (3), 26–32. 10.1111/j.1528-1167.2011.03033.x 21542843

[B2] Beal S. L. (2001). Ways to fit a PK model with some data below the quantification limit. J. Pharmacokinet. Pharmacodyn. 28, 481–504. 10.1023/a:1012299115260 11768292

[B3] Buchhalter J. R. D'Alfonso S. Connolly M. Fung E. Michoulas A. Sinasac D. (2017). The relationship between d-beta-hydroxybutyrate blood concentrations and seizure control in children treated with the ketogenic diet for medically intractable epilepsy. Epilepsia Open 2, 317–321. 10.1002/epi4.12058 29588960 PMC5862113

[B4] Buyken A. E. Goletzke J. Joslowski G. Felbick A. Cheng G. Herder C. (2014). Association between carbohydrate quality and inflammatory markers: systematic review of observational and interventional studies. Am. J. Clin. Nutr. 99, 813–833. 10.3945/ajcn.113.074252 24552752

[B5] Cervenka M. C. Hartman A. L. Venkatesan A. Geocadin R. G. Kossoff E. H. (2011). The ketogenic diet for medically and surgically refractory status epilepticus in the neurocritical care unit. Neurocrit Care 15, 519–524. 10.1007/s12028-011-9546-3 21523523

[B6] Cervenka M. C. Hocker S. Koenig M. Bar B. Henry-Barron B. Kossoff E. H. (2017). Phase I/II multicenter ketogenic diet study for adult superrefractory status epilepticus. Neurology 88, 938–943. 10.1212/WNL.0000000000003690 28179470 PMC5333514

[B7] Cervenka M. C. Kossoff E. H. (2013). Dietary treatment of intractable epilepsy. Contin. (Minneap Minn) 19, 756–766. 10.1212/01.CON.0000431396.23852.56 PMC10563885 23739109

[B8] De Simoni M. G. Perego C. Ravizza T. Moneta D. Conti M. Marchesi F. (2000). Inflammatory cytokines and related genes are induced in the rat hippocampus by limbic status epilepticus. Eur. J. Neurosci. 12, 2623–2633. 10.1046/j.1460-9568.2000.00140.x 10947836

[B9] DeLorenzo R. J. Hauser W. A. Towne A. R. Boggs J. G. Pellock J. M. Penberthy L. (1996). A prospective, population-based epidemiologic study of status epilepticus in Richmond, Virginia. Neurology 46, 1029–1035. 10.1212/wnl.46.4.1029 8780085

[B10] Deora V. Albornoz E. A. Zhu K. Woodruff T. M. Gordon R. (2017). The ketone body beta-hydroxybutyrate does not inhibit synuclein mediated inflammasome activation in microglia. J. Neuroimmune Pharmacol. 12, 568–574. 10.1007/s11481-017-9754-5 28836226

[B11] Devinsky O. Vezzani A. Najjar S. De Lanerolle N. C. Rogawski M. A. (2013). Glia and epilepsy: excitability and inflammation. Trends Neurosci. 36, 174–184. 10.1016/j.tins.2012.11.008 23298414

[B12] Dupuis N. Curatolo N. Benoist J. F. Auvin S. (2015). Ketogenic diet exhibits anti-inflammatory properties. Epilepsia 56, e95–e98. 10.1111/epi.13038 26011473

[B13] Eriksson C. Tehranian R. Iverfeldt K. Winblad B. Schultzberg M. (2000). Increased expression of mRNA encoding interleukin-1beta and caspase-1, and the secreted isoform of interleukin-1 receptor antagonist in the rat brain following systemic kainic acid administration. J. Neurosci. Res. 60, 266–279. 10.1002/(SICI)1097-4547(20000415)60:2<266::AID-JNR16>3.0.CO;2-P 10740232

[B14] Fabene P. F. Bramanti P. Constantin G. (2010). The emerging role for chemokines in epilepsy. J. Neuroimmunol. 224, 22–27. 10.1016/j.jneuroim.2010.05.016 20542576

[B15] Foresti M. L. Arisi G. M. Katki K. Montanez A. Sanchez R. M. Shapiro L. A. (2009). Chemokine CCL2 and its receptor CCR2 are increased in the hippocampus following pilocarpine-induced status epilepticus. J. Neuroinflammation 6, 40. 10.1186/1742-2094-6-40 20034406 PMC2804573

[B16] Francis B. A. Fillenworth J. Gorelick P. Karanec K. Tanner A. (2019). The feasibility, safety and effectiveness of a ketogenic diet for refractory status epilepticus in adults in the intensive care unit. Neurocrit Care 30, 652–657. 10.1007/s12028-018-0653-2 30484010

[B17] Frasch S. C. Bratton D. L. (2012). Emerging roles for lysophosphatidylserine in resolution of inflammation. Prog. Lipid Res. 51, 199–207. 10.1016/j.plipres.2012.03.001 22465125 PMC3365616

[B18] Fraser D. A. Thoen J. Bondhus S. Haugen M. Reseland J. E. Djoseland O. (2000a). Reduction in serum leptin and IGF-1 but preserved T-lymphocyte numbers and activation after a ketogenic diet in rheumatoid arthritis patients. Clin. Exp. Rheumatol. 18, 209–214. 10812493

[B19] Fraser D. A. Thoen J. Djoseland O. Forre O. Kjeldsen-Kragh J. (2000b). Serum levels of interleukin-6 and dehydroepiandrosterone sulphate in response to either fasting or a ketogenic diet in rheumatoid arthritis patients. Clin. Exp. Rheumatol. 18, 357–362. 10895373

[B20] Hocker S. Tatum W. O. LaRoche S. Freeman W. D. (2014). Refractory and super-refractory status epilepticus--an update. Curr. Neurol. Neurosci. Rep. 14, 452. 10.1007/s11910-014-0452-x 24760477

[B21] Hung Y. W. Lai M. T. Tseng Y. J. Chou C. C. Lin Y. Y. (2013). Monocyte chemoattractant protein-1 affects migration of hippocampal neural progenitors following status epilepticus in rats. J. Neuroinflammation 10, 11. 10.1186/1742-2094-10-11 23339567 PMC3563591

[B22] Jayalakshmi S. Ruikar D. Vooturi S. Alladi S. Sahu S. Kaul S. (2014). Determinants and predictors of outcome in super refractory status epilepticus--a developing country perspective. Epilepsy Res. 108, 1609–1617. 10.1016/j.eplepsyres.2014.08.010 25246354

[B23] Kan A. A. van der Hel W. S. Kolk S. M. Bos I. W. Verlinde S. A. van Nieuwenhuizen O. (2012). Prolonged increase in rat hippocampal chemokine signalling after status epilepticus. J. Neuroimmunol. 245, 15–22. 10.1016/j.jneuroim.2012.01.012 22353418

[B24] Kim do Y. Hao J. Liu R. Turner G. Shi F. D. Rho J. M. (2012). Inflammation-mediated memory dysfunction and effects of a ketogenic diet in a murine model of multiple sclerosis. PLoS One 7, e35476. 10.1371/journal.pone.0035476 22567104 PMC3342287

[B25] Knake S. Rosenow F. Vescovi M. Oertel W. H. Mueller H. H. Wirbatz A. (2001). Incidence of status epilepticus in adults in Germany: a prospective, population-based study. Epilepsia 42, 714–718. 10.1046/j.1528-1157.2001.01101.x 11422324

[B26] Li R. Ma L. Huang H. Ou S. Yuan J. Xu T. (2017). Altered expression of CXCL13 and CXCR5 in intractable temporal lobe epilepsy patients and pilocarpine-induced epileptic rats. Neurochem. Res. 42, 526–540. 10.1007/s11064-016-2102-y 27873133

[B27] Librizzi L. Noe F. Vezzani A. de Curtis M. Ravizza T. (2012). Seizure-induced brain-borne inflammation sustains seizure recurrence and blood-brain barrier damage. Ann. neurology 72, 82–90. 10.1002/ana.23567 22829270

[B28] Lin J. J. Lin K. L. Chan O. W. Hsia S. H. Wang H. S. CHEESE Study Group (2015). Intravenous ketogenic diet therapy for treatment of the acute stage of super-refractory status epilepticus in a pediatric patient. Pediatr. Neurol. 52, 442–445. 10.1016/j.pediatrneurol.2014.12.008 25771999

[B29] Lv R. Xu X. Luo Z. Shen N. Wang F. Zhao Y. (2014). Pyrrolidine dithiocarbamate (PDTC) inhibits the overexpression of MCP-1 and attenuates microglial activation in the hippocampus of a pilocarpine-induced status epilepticus rat model. Exp. Ther. Med. 7, 39–45. 10.3892/etm.2013.1397 24348761 PMC3861516

[B30] Mikati M. A. Abi‐Habib R. J. El Sabban M. E. Dbaibo G. S. Kurdi R. M. Kobeissi M. (2003). Hippocampal programmed cell death after status epilepticus: evidence for NMDA‐receptor and ceramide‐mediated mechanisms. Epilepsia 44, 282–291. 10.1046/j.1528-1157.2003.22502.x 12614382

[B31] Nam S. H. Lee B. L. Lee C. G. Yu H. J. Joo E. Y. Lee J. (2011). The role of ketogenic diet in the treatment of refractory status epilepticus. Epilepsia 52, e181–e184. 10.1111/j.1528-1167.2011.03289.x 22003821

[B32] Park E. G. Lee J. Lee J. (2019). The ketogenic diet for super-refractory status epilepticus patients in intensive care units. Brain Dev. 41, 420–427. 10.1016/j.braindev.2018.12.007 30638692

[B33] Peng P. Peng J. Yin F. Deng X. Chen C. He F. (2019). Ketogenic diet as a treatment for super-refractory status epilepticus in febrile infection-related epilepsy syndrome. Front. Neurol. 10, 423. 10.3389/fneur.2019.00423 31105638 PMC6498987

[B34] Pernot F. Heinrich C. Barbier L. Peinnequin A. Carpentier P. Dhote F. (2011). Inflammatory changes during epileptogenesis and spontaneous seizures in a mouse model of mesiotemporal lobe epilepsy. Epilepsia 52, 2315–2325. 10.1111/j.1528-1167.2011.03273.x 21955106

[B35] Ravizza T. Balosso S. Vezzani A. (2011). Inflammation and prevention of epileptogenesis. Neurosci. Lett. 497, 223–230. 10.1016/j.neulet.2011.02.040 21362451

[B36] Ravizza T. Vezzani A. (2006). Status epilepticus induces time-dependent neuronal and astrocytic expression of interleukin-1 receptor type I in the rat limbic system. Neuroscience 137, 301–308. 10.1016/j.neuroscience.2005.07.063 16289587

[B37] Rossetti A. O. Lowenstein D. H. (2011). Management of refractory status epilepticus in adults: still more questions than answers. Lancet Neurol. 10, 922–930. 10.1016/S1474-4422(11)70187-9 21939901 PMC3202016

[B38] Schreck K. C. Lwin M. Strowd R. E. Henry-Barron B. J. Blakeley J. O. Cervenka M. C. (2017). Effect of ketogenic diets on leukocyte counts in patients with epilepsy. Nutr. Neurosci. 22, 522–527. 10.1080/1028415x.2017.1416740 29254457

[B39] Strzelczyk A. Ansorge S. Hapfelmeier J. Bonthapally V. Erder M. H. Rosenow F. (2017). Costs, length of stay, and mortality of super-refractory status epilepticus: a population-based study from Germany. Epilepsia 58, 1533–1541. 10.1111/epi.13837 28681418

[B40] Strzelczyk A. Reif P. S. Bauer S. Belke M. Oertel W. H. Knake S. (2013). Intravenous initiation and maintenance of ketogenic diet: proof of concept in super-refractory status epilepticus. Seizure 22, 581–583. 10.1016/j.seizure.2013.03.007 23597842

[B41] Thakur K. T. Probasco J. C. Hocker S. E. Roehl K. Henry B. Kossoff E. H. (2014). Ketogenic diet for adults in super-refractory status epilepticus. Neurology 82, 665–670. 10.1212/WNL.0000000000000151 24453083 PMC3945660

[B42] Vezzani A. Aronica E. Mazarati A. Pittman Q. J. (2013). Epilepsy and brain inflammation. Exp. Neurol. 244, 11–21. 10.1016/j.expneurol.2011.09.033 21985866

[B43] Vezzani A. Granata T. (2005). Brain inflammation in epilepsy: experimental and clinical evidence. Epilepsia 46, 1724–1743. 10.1111/j.1528-1167.2005.00298.x 16302852

[B44] Wheeler D. Knapp E. Bandaru V. V. Wang Y. Knorr D. Poirier C. (2009). Tumor necrosis factor-alpha-induced neutral sphingomyelinase-2 modulates synaptic plasticity by controlling the membrane insertion of NMDA receptors. J. Neurochem. 109, 1237–1249. 10.1111/j.1471-4159.2009.06038.x 19476542 PMC2688711

[B45] Wusthoff C. J. Kranick S. M. Morley J. F. Christina Bergqvist A. G. (2009). The ketogenic diet in treatment of two adults with prolonged nonconvulsive status epilepticus. Epilepsia 51, 1083–1085. 10.1111/j.1528-1167.2009.02388.x 19845731

[B46] Yamanashi T. Iwata M. Kamiya N. Tsunetomi K. Kajitani N. Wada N. (2017). Beta-hydroxybutyrate, an endogenic NLRP3 inflammasome inhibitor, attenuates stress-induced behavioral and inflammatory responses. Sci. Rep. 7, 7677. 10.1038/s41598-017-08055-1 28794421 PMC5550422

[B47] Yang X. Cheng B. (2010). Neuroprotective and anti-inflammatory activities of ketogenic diet on MPTP-induced neurotoxicity. J. Mol. Neurosci. 42, 145–153. 10.1007/s12031-010-9336-y 20333481

[B48] Youm Y. H. Nguyen K. Y. Grant R. W. Goldberg E. L. Bodogai M. Kim D. (2015). The ketone metabolite beta-hydroxybutyrate blocks NLRP3 inflammasome-mediated inflammatory disease. Nat. Med. 21, 263–269. 10.1038/nm.3804 25686106 PMC4352123

[B49] Zhu X. B. Wang Y. B. Chen O. Zhang D. Q. Zhang Z. H. Cao A. H. (2012). Characterization of the expression of macrophage inflammatory protein-1α (MIP-1α) and C-C chemokine receptor 5 (CCR5) after kainic acid-induced status epilepticus (SE) in juvenile rats. Neuropathology Appl. Neurobiol. 38, 602–616. 10.1111/j.1365-2990.2012.01251.x 22248156

